# Associations between gestational weight gain and adverse pregnancy outcomes: a prospective cohort study

**DOI:** 10.7189/jogh.16.04375

**Published:** 2026-11-13

**Authors:** Xueyin Wang, Xiaosong Zhang, Meihua Zhang, Xu Chen, Xietong Wang, Yuyan Ma, Yue Teng, Haixia Meng, Xiaoqing Wang, Qiuhong Yang, Lin Xu, Shufan Shan, Juan Juan, Di Gao, Huixia Yang

**Affiliations:** 1 Department of Obstetrics and Gynaecology and Reproductive Medicine, Peking University First Hospital, Beijing, China; 2 Department of Obstetrics and Gynaecology, Taiyuan Maternity and Child Health Hospital, Taiyuan, China; 3 Department of Obstetrics, Tianjin Central Obstetrics and Gynaecology Hospital, Tianjin, China; 4 Department of Obstetrics and Gynaecology, Shandong Provincial Hospital Affiliated with Shandong First Medical University, Jinan, China; 5 Department of Obstetrics and Gynaecology, Qilu Hospital of Shandong University, Jinan, China; 6 Department of Nutrition, Haidian District Maternal and Child Health Care Hospital, Beijing, China; 7 Department of Obstetrics and Gynaecology, The Affiliated Hospital of Inner Mongolia Medical University, Hohhot, China; 8 Department of Obstetrics and Gynaecology, The Third People’s Hospital of Datong, Datong, China; 9 Department of Obstetrics, Jinan Maternity and Child Care Hospital, Jinan, China; 10 Department of Obstetrics and Gynaecology, The Affiliated Hospital of Qingdao University, Qingdao, China; 11 Department of Obstetrics and Gynaecology, Affiliated Hospital of Chifeng University, Chifeng, China

**Keywords:** gestational weight gain, caesarean section, small for gestational age, large for gestational age, macrosomia

## Abstract

**Background:**

Most studies on gestational weight gain (GWG) and pregnancy outcomes have focused on Western populations, leaving a gap in knowledge about the applicability of their findings to Asian women. Similarly, most investigations have utilised recommendations created by the US Institute of Medicine, with few using the Chinese GWG recommendations. We aimed to assess the associations between GWG, evaluated according to the Chinese recommendations, and adverse pregnancy outcomes, as well as to explore whether prepregnancy body mass index (BMI) or maternal age modified these associations.

**Methods:**

In this prospective cohort study, we recruited pregnant women at 11–13 weeks of gestation from 13 hospitals in northern China between October 2020 and June 2024. We categorised GWG as insufficient, adequate, or excessive when it fell below, within, or above the Chinese recommendations, with the recommended GWG ranges being 11.0–16.0 kg (BMI < 18.5 kg/m^2^), 8.0–14.0 kg (18.5–23.9 kg/m^2^), 7.0–11.0 kg (24.0–27.9 kg/m^2^), and 5.0–9.0 kg (BMI ≥ 28.0 kg/m^2^). We used logistic regression models to estimate odds ratios (ORs) for adverse pregnancy outcomes.

**Results:**

Among 10,638 pregnant women, 13.3%, 54.3%, and 32.4% had insufficient, adequate, and excessive GWG, respectively. After adjusting for confounders, excessive GWG was associated with higher odds for caesarean delivery (OR = 1.38; 95% confidence interval (CI) = 1.26–1.53), macrosomia (OR = 1.77; 95% CI = 1.44–2.17), being large for gestational age (OR = 1.66; 95% CI = 1.38–1.99), and neonatal ward admission (OR = 1.35; 95% CI = 1.09–1.67), while insufficient GWG was associated with increased odds of being small for gestational age (OR = 1.48; 95% CI = 1.17–1.88). There was a significant interaction between GWG and maternal age in relation to the odds of caesarean delivery (*P*-value for interaction = 0.034). Women aged ≥ 35 years with excessive GWG had the highest odds of caesarean delivery (OR = 2.72; 95% CI = 2.27–3.24), compared with women aged <35 years with adequate GWG.

**Conclusions:**

Excessive GWG was associated with higher odds of caesarean delivery, macrosomia, large for gestational age, and neonatal ward admission, while insufficient GWG was associated to higher odds of small for gestational age.

Gestational weight gain (GWG) has an important and independent impact on both maternal and neonatal health, regardless of the maternal prepregnancy body mass index (BMI) [1]. A recent systematic review and meta-analysis encompassing more than 1.6 million pregnancies worldwide indicated that excessive GWG affected 45% of pregnancies, while insufficient GWG affected 23% [2]. Excessive GWG has been linked to increased risks of gestational diabetes mellitus (GDM), caesarean delivery, macrosomia, and the delivery of large-for-gestational age (LGA) infants [1,3]. Conversely, insufficient GWG raises the risks of preterm birth, low birth weight, the delivery of small-for-gestational-age (SGA) infants, nutritional deficiency, and neonatal intensive care unit admissions [1,3,4]. In the longer term, inadequate GWG could also be correlated with postpartum depression, weight retention, maternal cardiometabolic risk, and cognitive impairment in offspring [5].

In general, GWG reflects a complex interaction among multiple factors, mainly nutritional status during pregnancy, foetal growth, amniotic fluid, and fluid retention [2]. As it is one of the modifiable influential factors for pregnancy outcomes, researchers have extensively investigated what the optimal GWG would be and have developed recommendations for different ethnic populations to promote maternal and neonatal health [6,7]. For instance, the US Institute of Medicine (IoM) issued updated recommendations for weight gain during pregnancy in 2009 based on data from pregnant women in the USA [6], which have been widely utilised by population-based studies conducted on predominantly Western populations. However, research based on these guidelines conducted in other contexts has reported inconsistent findings: while studies conducted in North America, Europe, and South Asia revealed a significant positive association between excessive GWG and macrosomia [8–10], a systematic review and meta-analysis found no such association among sub-Saharan African populations [11]. Previous research has also been constrained by methodological limitations such as small sample sizes, single-centre designs, and inadequate adjustment for major confounders such as prepregnancy BMI, ethnicity, and gestational age at delivery, which reduces the generalisability and validity of its findings.

Differences in body composition, fat distribution, lifestyle, and genetic factors between Asian and US females have raised concerns about the applicability of the IoM recommendations to Asian populations [12–14]. For this reason, the National Health Commission of the People’s Republic of China released the standard of recommendations (hereafter referred to as ‘Chinese recommendations’) in 2022, which provides BMI-specific GWG recommendations based on data from pregnant women in nine cities in China [7]. While these recommendations have since been commonly adopted in clinical practice in China, their applicability to the country’s population remains unclear, as few studies have used them to examine the associations between GWG and pregnancy outcomes. To address this gap, we designed a multicentre prospective cohort study that would apply the latest Chinese recommendations to assess the associations between GWG below and above the recommended ranges and the risks of six adverse pregnancy outcomes: caesarean delivery, low birth weight, SGA, macrosomia, LGA, and neonatal ward admission. We selected these primary outcomes because they collectively cover the full foetal growth spectrum, are relevant to national surveillance, and have well-established definitions that facilitate comparisons with previous studies. We also examined whether prepregnancy BMI or maternal age modified these associations.

## METHODS

### Study design and sites

We conducted this prospective, observational cohort study from October 2020 to June 2024 at 13 tertiary hospitals across seven provincial-level administrative divisions of northern China: Beijing Municipality, Tianjin Municipality, Shanxi Province, Hebei Province, Shaanxi Province, Shandong Province, and Inner Mongolia Autonomous Region. All hospitals had standardised electronic medical record systems and routine obstetric data collection protocols.

### Participants

We enrolled pregnant women at 11–13 weeks of gestation at the participating hospitals. We included participants aged ≥18 years who planned to receive antenatal care and be hospitalised for delivery at the participating hospitals. We excluded participants who had severe chronic disease before pregnancy and had severe mental illness.

### Data collection

We followed up all enrolled women during the second and third trimesters and after delivery. Investigators initially conducted face-to-face interviews with each participant at enrolment to collect information on sociodemographic characteristics, lifestyle factors, and medical history. This included data on maternal age, ethnicity (Han or other), education (junior school and below, senior high school, or college or graduate school), employment (employed or unemployed), average monthly household income (RMB < 3,000, 3,000–5,000, 5,000–10,000, and >10,000), alcohol consumption during pregnancy (yes or no), smoking before pregnancy (yes or no), passive smoking during pregnancy (yes or no), and parity (0, 1 and ≥2). We obtained information on gestational weeks at delivery, GDM, and hypertension in pregnancy from the participants’ medical records.

### Measurement and classification of prepregnancy BMI and GWG

We measured the participants’ weight and height while they wore light clothing at the first antenatal care visit before 13 weeks of gestation. We calculated prepregnancy BMI as weight in kilograms divided by height meters squared, with participants classified according to the cut-offs recommended by the Working Group on Obesity in China as underweight (BMI < 18.5 kg/m^2^), normal weight (BMI = 18.5–23.9 kg/m^2^), overweight (BMI = 24.0–27.9 kg/m^2^) and obese (BMI ≥ 28.0 kg/m^2^) [15]. We determined the GWG as the difference between the weight measured at the last antenatal care visit before delivery and that measured at the first antenatal care visit, and classified it according to the Chinese recommendations as insufficient, adequate, or excessive if it was below, within, or above the recommendation, respectively, as follows: 11.0–16.0 kg (underweight), 8.0–14.0 kg (normal weight), 7.0–11.0 kg (overweight), and 5.0–9.0 kg (obese) [7].

In further sensitivity analyses based on the 2009 IoM recommendations [16], we classified GWG as insufficient, adequate, and excessive if it was below, within, or above the following ranges: 12.5–18 kg (prepregnancy BMI < 18.5 kg/m^2^), 11.5–16 kg (prepregnancy BMI = 18.5–24.9 kg/m^2^), 7–11.5 kg (prepregnancy BMI = 25.0–29.9 kg/m^2^), and 5–9 kg (prepregnancy BMI ≥ 30.0 kg/m^2^). We also classified the weekly GWG rate during the second and third trimesters according to the Chinese recommendations as insufficient, adequate and excessive if it was below, within, or above the range, respectively: 0.37–0.56 kg/week for a prepregnancy BMI < 18.5 kg/m^2^, 0.26–0.48 kg/week for a prepregnancy BMI of 18.5–23.9 kg/m^2^, 0.22–0.37 kg/week for a prepregnancy BMI of 24.0–27.9 kg/m^2^, and 0.15–0.30 kg/week for a prepregnancy BMI ≥ 28.0 kg/m^2^ [7].

### Assessment and definition of perinatal outcomes

Using data retrieved from medical records, we assessed six adverse pregnancy outcomes: caesarean delivery, low birth weight, SGA, macrosomia, LGA, and neonatal ward admission. We defined low birth weight as birth weight <2,500 g and macrosomia as birth weight ≥4,000 g. We defined SGA and LGA as birth weights below the sex- and gestational age-specific 10th percentile or above the 90th, respectively, according to the Chinese neonatal birth weight reference curve [17].

### Statistical analysis

We summarised baseline characteristics and pregnancy outcomes as medians and interquartile ranges for skewed continuous variables and as numbers and percentages for categorical variables. We compared the former using Mann-Whitney U tests and the latter using χ^2^ tests. We used univariate and multivariate logistic regression models to assess pregnancy outcomes across GWG categories, with the outputs presented as odds ratios (ORs) and 95% confidence intervals (CIs). We adjusted multivariate models for hospitals, maternal age, prepregnancy BMI categories, education, employment, average household income, ethnicity, parity, smoking before pregnancy, passive smoking during pregnancy, alcohol consumption during pregnancy, and gestational age at delivery. We treated participants with appropriate GWG as the reference group.

We conducted stratified analyses according to maternal age (<35 or ≥35 years) and prepregnancy BMI category (underweight, normal weight, overweight, or obese), examining these interactions by including the cross-product term together with their main effects. We performed several sensitivity analyses on the associations between GWG and adverse pregnancy outcomes. First, additionally adjusted for GDM and hypertension in pregnancy. Second, we reclassified GWG according to the 2009 IoM recommendations. Third, we conducted the assessment using the GWG rate according to the Chinese recommendations. We performed all analyses using SAS, version 9.4 (SAS Institute, Cary, North Carolina, USA). All tests were two-sided, and we considered *P*-values <0.05 statistically significant.

## RESULTS

### Participant characteristics

Our sample included 10,638 pregnant women, of whom 13.3% had insufficient GWG, 54.3% had adequate GWG, and 32.4% had excessive GWG. Adequate GWG was most prevalent among women with normal prepregnancy BMI (60.4%). We observed excessive GWG in 43.4% of women who were overweight before pregnancy and 51.0% in women who were obese before pregnancy; both proportions were higher than those among women with normal prepregnancy BMI. Insufficient GWG was most prevalent among women who were underweight before pregnancy (28.5%). The distribution of GWG varied according to prepregnancy BMI (**Figure 1**).

**Figure 1:**
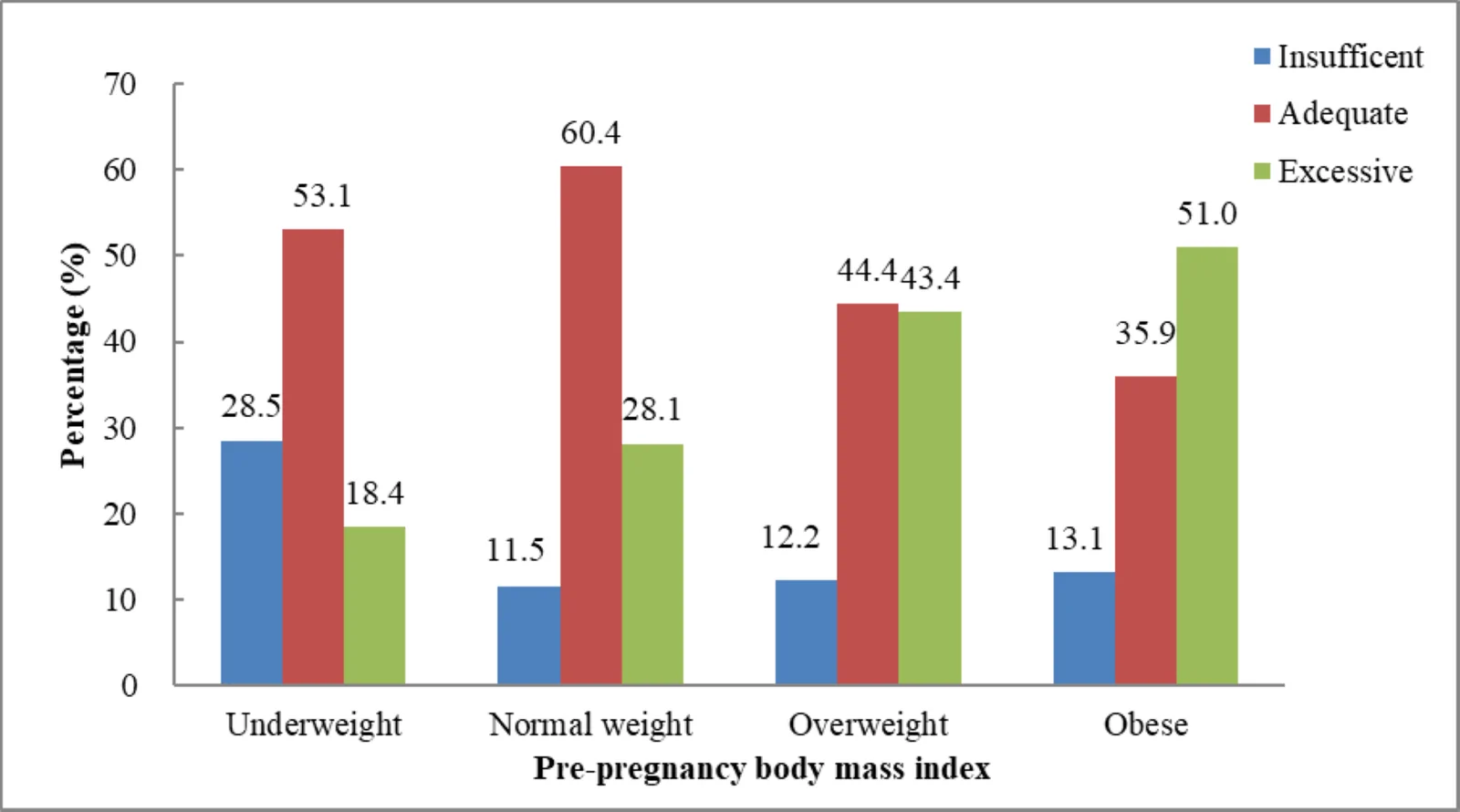
Distribution of gestational weight gain categories by prepregnancy body mass index groups.

Women with insufficient or excessive GWG were more likely to have a higher prepregnancy BMI (*P* < 0.001) and lower levels of education (*P* < 0.001), more likely to be parous (*P* < 0.001) and exposed to passive smoke during pregnancy (*P* < 0.001), and less likely to be employed (*P* = 0.001), compared to women with adequate GWG (*P* < 0.05). Women with excessive GWG tended to smoke before pregnancy and drink alcohol during pregnancy (*P* < 0.001), and had a higher rate of hypertension in pregnancy (*P* < 0.001), and a lower rate of GDM (*P* < 0.001), than women with adequate GWG (*P* < 0.05) (**Table 1**).

**Table 1:** Characteristics of study participants*

	Total (n = 10,638, 100.0%)	Insufficient (n = 1,413, 13.3%)	Adequate (n = 5,772, 54.3%)	Excessive (n = 3,453, 32.4%)	*P-*value
**Maternal age in years, Mdn (IQR)**	31 (28–34)	31 (29–34)	31 (29–34)	31 (28–34)	<0.001
**Prepregnancy BMI in kg/m^2^, Mdn (IQR)**	22.1 (20.1–24.5)	22.0 (19.5–24.4)	21.7 (20.0–23.7)	22.9 (20.7–25.7)	<0.001
**Gestational weight gain in kg, Mdn (IQR)**	12.0 (9.1–15.0)	6.0 (3.6–7.1)	11.0 (9.4–12.6)	16.0 (14.4–18.0)	<0.001
**Gestational age at delivery in weeks, Mdn (IQR)**	39 (39–40)	39 (39–40)	39 (39–39)	39 (39–40)	0.620
**Education**					<0.001
Junior school and below	415 (3.9)	57 (4.0)	214 (3.7)	144 (4.1)	
Senior high school	789 (7.4)	101 (7.2)	375 (6.5)	313 (9.1)	
College or graduate school	9,434 (88.7)	1,255 (88.8)	5,183 (89.8)	2,996 (86.8)	
**Employment**					0.002
Employed	9,370 (88.1)	1,225 (86.7)	5,144 (89.1)	3,001 (86.9)	
Unemployed	1,268 (11.9)	188 (13.3)	628 (10.9)	452 (13.1)	
**Average household income per month in RMB**					0.172
<3,000	336 (3.2)	51 (3.6)	170 (3.0)	115 (3.3)	
3,000–5,000	1,003 (9.4)	132 (9.3)	516 (8.9)	355 (10.3)	
5,000–10,000	3,476 (32.7)	450 (31.9)	1,880 (32.6)	1,146 (33.2)	
>10,000	5,823 (54.7)	780 (55.2)	3,206 (55.5)	1,837 (53.2)	
**Ethnicity**					0.348
Han	10,262 (96.5)	1,366 (96.7)	5,578 (96.6)	3,318 (96.1)	
Other	376 (3.5)	47 (3.3)	194 (3.4)	135 (3.9)	
**Parity**					<0.001
Primiparous	7,957 (74.8)	1,038 (73.5)	4,416 (76.5)	2,503 (72.5)	
Parous	2,681 (25.2)	352 (24.9)	1,250 (21.7)	848 (24.6)	
**Smoking before pregnancy**					<0.001
No	10,486 (98.6)	1,397 (98.9)	5,711 (98.9)	3,378 (97.8)	
Yes	152 (1.4)	16 (1.1)	61 (1.1)	75 (2.2)	
**Passive smoking during pregnancy**					<0.001
No	9,923 (93.3)	1,321 (93.5)	5,437 (94.2)	3,165 (91.7)	
Yes	715 (6.7)	92 (6.5)	335 (5.8)	288 (8.3)	
**Alcohol consumption during pregnancy**					<0.001
No	9,948 (93.5)	1,340 (94.8)	5,441 (94.3)	3,167 (91.7)	
Yes	690 (6.5)	73 (5.2)	331 (5.7)	286 (8.3)	
**Prepregnancy BMI category**					<0.001
Underweight	949 (8.9)	270 (19.1)	504 (8.7)	175 (5.1)	
Normal weight	6,550 (61.6)	752 (53.2)	3,956 (68.5)	1,842 (53.3)	
Overweight	2,179 (20.5)	265 (18.8)	967 (16.8)	947 (27.4)	
Obese	960 (9.0)	126 (8.9)	345 (6.0)	489 (14.2)	
**Gestational diabetes mellitus**					<0.001
No	8,516 (80.1)	1,019 (72.1)	4,620 (80.0)	2,877 (83.3)	
Yes	2,122 (19.9)	394 (27.9)	1,152 (20.0)	576 (16.7)	
**Hypertension during pregnancy**					<0.001
No	9,866 (92.7)	1,316 (93.1)	5,447 (94.4)	3,103 (89.9)	
Yes	772 (7.3)	97 (6.9)	325 (5.6)	350 (10.1)	

BMI – body mass index, IQR – interquartile range, Mdn – median

*Presented as n (%) unless specified otherwise.

### Distribution of pregnancy outcomes by GWG

Overall, GWG was positively associated with the risks of macrosomia and LGA (*P* < 0.001). Both insufficient and excessive GWG were associated with higher rates of caesarean delivery, SGA, and neonatal ward admission compared to adequate GWG (*P* < 0.05) (Table S1 in the **Online Supplementary Document**).

### Associations between GWG and pregnancy outcomes

After adjustment for potential confounders, excessive GWG was associated with higher odds of caesarean delivery (OR = 1.38; 95% CI = 1.26–1.53), macrosomia (OR = 1.77; 95% CI = 1.44–2.17), LGA (OR = 1.66; 95% CI = 1.38–1.99), and neonatal ward admission (OR = 1.35; 95% CI = 1.09–1.67). Insufficient GWG was associated with higher odds of SGA (OR = 1.48; 95% CI = 1.17–1.88) and lower odds of LGA (OR = 0.65; 95% CI = 0.47–0.89). For low birthweight, neither the unadjusted (insufficient: OR = 0.85; 95% CI = 0.52–1.38; excessive: OR = 0.73; 95% CI = 0.51–1.05) nor the adjusted model (insufficient: OR = 0.73, 95% CI = 0.44–1.20; excessive: OR = 0.76; 95% CI = 0.51–1.13) showed a statistically significant association with GWG (**Table 2**).

**Table 2:** Associations between gestational weight gain and pregnancy outcomes (n = 10,638), presented as OR (95% CI)*

	Insufficient	Adequate	Excessive
**Caesarean delivery**			
Model 1	1.07 (0.95–1.22)	ref	1.57 (1.43–1.71)
Model 2	0.95 (0.83–1.08)	ref	1.38 (1.26–1.53)
**Low birth weight**			
Model 1	0.85 (0.52–1.38)	ref	0.73 (0.51–1.05)
Model 2	0.73 (0.44–1.20)	ref	0.76 (0.51–1.13)
**SGA**			
Model 1	1.64(1.30–2.07)	ref	1.09(0.90–1.33)
Model 2	1.48(1.17–1.88)	ref	1.13(0.92–1.38)
**Macrosomia**			
Model 1	0.79 (0.56–1.12)	ref	2.10 (1.73–2.55)
Model 2	0.78 (0.55–1.11)	ref	1.77 (1.44–2.17)
**LGA**			
Model 1	0.74 (0.54–1.01)	ref	2.23 (1.88–2.64)
Model 2	0.65 (0.47–0.89)	ref	1.66 (1.38–1.99)
**Neonatal ward admission**			
Model 1	1.18 (0.90–1.56)	ref	1.37 (1.13–1.67)
Model 2	1.04 (0.77–1.39)	ref	1.35 (1.09–1.67)

CI – confidence interval, LGA – large for gestational age, OR – odds ratio, ref – reference, SGA – small for gestational age

*Model 1 was unadjusted. Model 2 was adjusted for hospital, maternal age, prepregnancy body mass index category, education, employment, average household income, ethnicity, parity, smoking before pregnancy, passive smoking during pregnancy, alcohol consumption during pregnancy, and gestational age at delivery.

### Stratified, interaction, and sensitivity analyses

Stratified analyses by prepregnancy BMI categories yielded results generally consistent with those observed in the overall population. Effect estimates varied slightly across BMI categories, but none of the interaction analyses reached statistical significance, with all *P*-values for interaction being >0.05 (Tables S2 and S3 in the **Online Supplementary Document**).

We observed a significant interaction between GWG and maternal age in relation to the odds of caesarean delivery in the univariate (*P*-value for interaction = 0.031) and multivariate logistic models (*P*-value for interaction = 0.034). Among women aged <35 years, the OR for the association between excessive GWG and the risk of caesarean delivery was 1.28 (95% CI = 1.15–1.43) after multiple adjustments. Women aged ≥35 years with excessive GWG had 78% higher odds of caesarean delivery compared with the reference group (OR = 1.78; 95% CI = 1.44–2.20).

We further performed a combined analysis to examine the effect of GWG and maternal age on caesarean delivery (**Figure 2**; Table S4 in the **Online Supplementary Document**). Women aged ≥35 years with excessive GWG had the highest risk of caesarean delivery (OR = 2.72; 95% CI = 2.27–3.24) compared with women aged <35 years with appropriate GWG. We observed no significant interaction between GWG and prepregnancy BMI on pregnancy outcomes.

**Figure 2:**
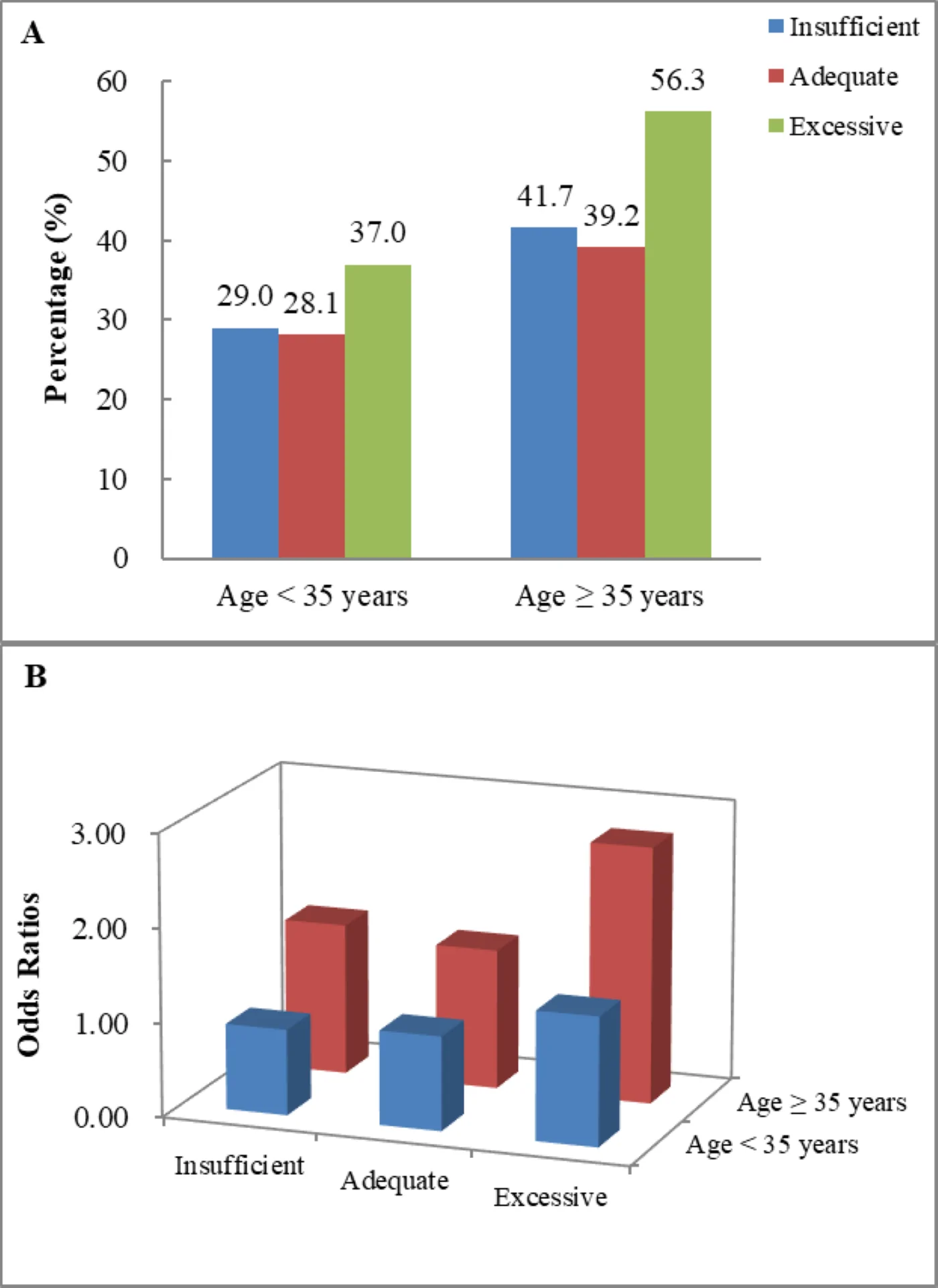
Associations of gestational weight gain and maternal age with the odds of caesarean delivery. **Panel A.** Frequencies and percentages of caesarean deliveries across gestational weight gain and maternal age groups. **Panel B.** Adjusted odds ratios for caesarean delivery across gestational weight gain and maternal age groups. The model was adjusted for hospital, prepregnancy body mass index category, education, employment, average household income, ethnicity, parity, smoking before pregnancy, passive smoking during pregnancy, alcohol consumption during pregnancy, and gestational age at delivery. GDM – gestational diabetes mellitus.

The absolute risk increase associated with advanced maternal age was largest in the adequate GWG group (absolute risk difference (ARD) = 16.75; 95% CI = 12.39–21.12, *P* < 0.001), followed by the insufficient GWG group (ARD = 12.50; 95% CI = 6.04–18.97, *P* < 0.001) and the excessive GWG group (ARD = 9.79; 95% CI = 6.34-13.24, *P* < 0.001) (Table S5 in the **Online Supplementary Document**). The results were generally consistent with those in the overall population.

We performed several sensitivity analyses on the associations between GWG and adverse pregnancy outcomes. First, additionally adjusted for GDM and hypertension in pregnancy. Second, we reclassified GWG according to the 2009 IoM recommendations. Third, we conducted the assessment using the GWG rate according to the Chinese recommendations (Tables S6–8 in the **Online Supplementary Document**). The results were generally consistent with those in the overall population.

## DISCUSSION

When categorised according to Chinese recommendations, more than half (54.3%) of the 10,638 pregnant Chinese women in our sample had adequate GWG, around a third (32.4%) had excessive GWG, and 13.3% had insufficient GWG. We found that excessive GWG was associated with increased odds of caesarean delivery, macrosomia, LGA, and neonatal ward admission, while insufficient GWG was associated with higher odds of SGA. We also found an interaction between GWG and maternal age in relation to the odds of caesarean delivery.

The IoM recommendations, updated in 2009, were developed using data from women in the USA and have been widely used in clinical practice and population-based studies in many countries for the past decade. Differences in body composition between Western and Asian females, together with the limited generalisability of the IoM recommendations to Asian populations [2,13], led to subsequent development and release of recommendations for weight gain during pregnancy based on data from Chinese women. In 2021, the Chinese Nutrition Society issued the recommendation entitled Weight Monitoring and Evaluation During Pregnancy Period of Chinese Women [18]. In 2022, the National Health Commission of the People’s Republic of China issued the health industry standard that provided the same recommended GWG ranges as the Chinese Nutrition Society and has been widely adopted in clinical practices and research in China [7].

Compared with previous studies conducted in Chinese populations applying the IoM recommendations, more women in our cohort were classified as having adequate GWG, fewer as having adequate GWG (27.4–42.7%), and fewer as having excessive GWG (33.9–50%) [12,15,19]. Our results also indicate that excessive GWG was associated with higher odds of macrosomia and LGA. A recent systematic review and meta-analysis encompassing more than 1.6 million pregnant women reported findings consistent with ours: GWG above the IoM recommendations was associated 77% and 78% elevated risk of LGA and macrosomia, respectively [3]. Several population-based studies among Chinese women also reported significant associations between GWG above the IoM recommended ranges and the risk of LGA and macrosomia [19–21], which aligns with the results of our analysis, which utilised the Chinese recommendations. Excessive GWG is known to be closely correlated with abnormal variations in glucose and lipid metabolism and increased insulin resistance, which may affect placental function, foetal insulin sensitivity, and the foetal metabolic environment, thereby promoting foetal fat accumulation and higher birth weight [22].

We also found that excessive GWG was associated with an increased risk of neonatal ward admission. The aforementioned meta-analysis reported a similar finding, whereby GWG above the IoM-recommended ranges was associated with a 1.26-fold higher risk of neonatal intensive care unit admission [2]. A recent population-based retrospective cohort study of 18,575 Iranian women reported that excessive GWG was associated with 86% higher odds of neonatal intensive care unit admission [23]. Moreover, a secondary analysis of 8,628 nulliparous women also found a higher rate of neonatal intensive care unit admission among neonates born to women with excessive GWG (14.3%) than among those born to women with adequate GWG in the overall cohort (13.6%) [24], which may partly support our results. One plausible explanation is that excessive GWG contributes to higher risks of GDM, macrosomia, and LGA, which might lead to hypoglycaemia, birth trauma, and distress for neonates and further increase the likelihood of neonatal ward admission. However, since neonatal ward admissions are affected by various correlated factors beyond GWG, further research is required to clarify the underlying mechanisms.

We also found an association between insufficient GWG and increased odds of SGA. Similarly, several previous studies conducted in different ethnic populations suggested that women with inadequate GWG according to the IoM-recommended ranges had an elevated risk of SGA [20,21,25,26]. We further observed that insufficient GWG was associated with an increased risk of SGA only among women who were underweight or normal weight before pregnancy, which aligns with the findings of a retrospective cohort study conducted on Taipei women [20]. Previous evidence has indicated that GWG above the IoM recommendations was associated with a lower risk of SGA [2]. However, we did not identify this association in our study, possibly because of differences in study design, GWG category cut-offs, and the ethnic groups studied.

We identified a significant interaction between GWG and maternal age in relation to the odds of caesarean delivery. Specifically, women aged ≥35 years with excessive GWG had nearly three times the odds of caesarean delivery as women aged below 35 years with adequate GWG. Within the group aged ≥35 years, women with excessive GWG had 1.78-fold higher odds of caesarean delivery than those with appropriate GWG. These findings suggest that excessive GWG and maternal age ≥35 years jointly increase the odds of caesarean delivery. Consistent with these results, a previous retrospective cohort study of 2,495 women with term pregnancies reported that the risk of caesarean delivery increased by 13% for each 5 kg increment in GWG and by 5% for each year of maternal age [27]. Moreover, earlier evidence demonstrated associations between excessive GWG and advanced maternal age and a greater risk of caesarean delivery [28–30]. One plausible interpretation is that uterine ageing is closely related to reduced uterine contractility performance through alterations in intracellular factors and signalling pathways, such as intracellular calcium regulation, electrical conductance, and mitochondrial function [31]. Furthermore, advanced age has been linked to a heightened risk of comorbidities, including GDM and hypertensive disorders of pregnancy, which excessive GWG may exacerbate [32]. In addition, the synergistic effects of uterine ageing and increased foetal size and likelihood of labour dystocia associated with excessive GWG may collectively contribute to a higher incidence of caesarean delivery [33].

Our findings highlight the importance of age-stratified GWG management, particularly for women aged ≥35 years. Incorporating age-specific GWG targets into routine antenatal care may help reduce the incidence of caesarean deliveries among the high-risk populations because advanced maternal age and excessive GWG carry a compounded risk. Notably, a balanced approach addressing both insufficient and excessive GWG remains essential in clinical practice and future research, as interventions targeting excessive gain should be carefully weighed against the risk of SGA and inadequate foetal growth.

This study has several strengths, including its prospective design, relatively large sample size, and adjustments for multiple potential confounders, all of which enhance the statistical power, robustness, and validity of the obtained results. In addition, our analysis extends the existing evidence by providing a significant interaction between excessive GWG and advanced maternal age in relation to the risk of caesarean delivery. This finding emphasises the clinical importance of maintaining adequate GWG, particularly among pregnant women aged ≥35 years.

However, several limitations should be mentioned. First, our hospital-based sample consisted exclusively of Chinese Han women with higher sociodemographic status, which may limit the generalisability of our findings to other ethnic populations, geographic regions, or healthcare settings without external validation. Second, we used first-trimester weight to calculate prepregnancy BMI, which may introduce nondifferential misclassification due to early-pregnancy weight gain, potentially attenuating the true associations toward the null. Third, data on several potential confounders, including previous caesarean delivery, assisted reproductive technology, diet, physical activity, and sleep, were unavailable in this study. We were, therefore, unable to adjust for these factors, which may have introduced residual confounding into the observed associations between GWG and pregnancy outcomes. Moreover, the outcomes of caesarean delivery and neonatal ward admission were susceptible to non-biological influences, such as clinical preference and local practices. The lack of detailed data on indications for caesarean delivery and reasons for neonatal ward admission limited our ability to distinguish between biologically driven effects and practice-related variation. Finally, although the number of excluded women was small, systematic differences in health-seeking behaviours or socioeconomic status may have introduced selection bias, possibly resulting in a healthier analytic sample and thus producing conservative estimates. Future well-designed prospective studies are warranted to confirm our findings.

## CONCLUSIONS

We found that excessive GWG was associated with increased odds of caesarean delivery, macrosomia, LGA, and neonatal ward admission, while insufficient GWG was associated with higher odds of SGA. We also identified a significant interaction between GWG and maternal age in relation to the odds of caesarean delivery. These results highlight the importance of maintaining GWG within recommended guidelines, particularly for women aged ≥35 years. Balanced clinical and research strategies addressing both insufficient and excessive GWG are essential to improve maternal and neonatal outcomes.

## Additional material


Online Supplementary Document

## Data Availability

**Data availability:** The data are available from the corresponding authors upon request and subject to their approval.
