# Supplementary material for: Associations between gestational weight gain and adverse pregnancy outcomes: a prospective cohort study
Source: J Glob Health. 2026 Nov 13;16:04375. doi: 10.7189/jogh.16.04375 (PMC13761197; doi:10.7189/jogh.16.04375)
Supplement: Supplementary file 1 [file jogh-16-04375-s001.pdf]

Supplement to: Wang X, Zhang X, Juan J, Gao D, Yang H, Zhang M, Chen X, Wang X, Ma Y, Teng Y, Meng H, Wang X, Yang Q, Xu L, Shan S. Associations between gestational weight gain and adverse pregnancy outcomes: a prospective cohort study. J Glob Health. 2026;16:04375.

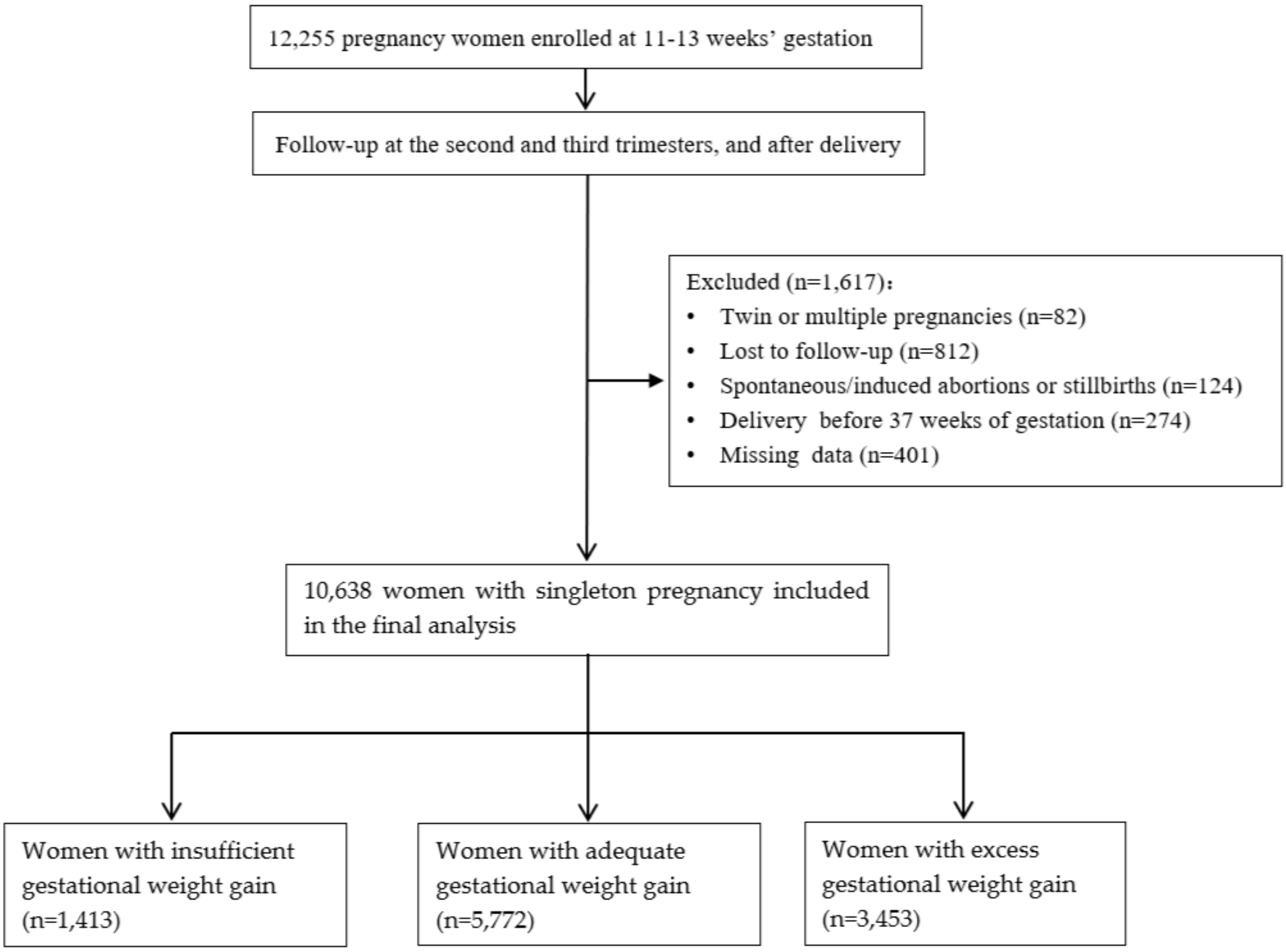

Figure S1 1. Flow chart of the study

Table S1. Adverse pregnancy outcomes of study participants (n=10,638)

	Total	Insufficient	Adequate	Excess	*P* value
Cesarean delivery	3626 (34.1)	453 (32.1)	1764 (30.6)	1409 (40.8)	<0.001
Low birthweight	158 (1.5)	20 (1.4)	96 (1.7)	42 (1.2)	0.223
SGA	551 (5.2)	105 (7.4)	270 (4.7)	176 (5.1)	<0.001
Macrosomia	481 (4.5)	39 (2.8)	200 (3.5)	242 (7.0)	<0.001
LGA	629 (5.9)	47 (3.3)	257 (4.5)	325 (9.4)	<0.001
Neonatal ward admission	503 (4.7)	69 (4.9)	240 (4.2)	194 (5.6)	0.006

Data are presented as n (%).

Table S2. Association between gestational weight gain and pregnancy outcomes by prepregnancy body mass index

Outcome	Insufficient	Adequate	Excess	*P* for interaction
Cesarean delivery				0.288
Underweight	0.77 (0.53, 1.12)	Reference	1.34 (0.89, 2.03)	
Normal weight	0.99 (0.83, 1.18)	Reference	1.54 (1.35, 1.74)	
Overweight	0.82 (0.61, 1.11)	Reference	1.15 (0.94, 1.41)	
Obese	1.09 (0.70, 1.70)	Reference	1.20 (0.87, 1.64)	
Low birthweight				0.357
Underweight	0.60 (0.20, 1.81)	Reference	0.16 (0.02, 1.38)	
Normal weight	0.77 (0.36, 1.65)	Reference	1.10 (0.67, 1.82)	
Overweight	0.38 (0.08, 1.75)	Reference	0.40 (0.16, 1.00)	
Obese	0.82 (0.19, 3.62)	Reference	0.70 (0.23, 2.13)	
SGA				0.854
Underweight	1.67 (1.03, 2.73)	Reference	1.02 (0.54, 1.94)	
Normal weight	1.40 (1.00, 1.97)	Reference	1.23 (0.95, 1.59)	
Overweight	1.36 (0.73, 2.54)	Reference	0.91 (0.58, 1.44)	
Obese	1.63 (0.70, 3.80)	Reference	1.00 (0.52, 1.92)	
Macrosomia				0.415
Underweight	1.03 (0.18, 5.95)	Reference	2.28 (0.47, 11.12)	
Normal weight	1.11 (0.71, 1.73)	Reference	1.91 (1.44, 2.52)	
Overweight	0.32 (0.13, 0.75)	Reference	1.47 (1.01, 2.14)	
Obese	0.85 (0.33, 2.21)	Reference	1.64 (0.92, 2.92)	
LGA				0.634
Underweight	0.59 (0.15, 2.26)	Reference	1.76 (0.55, 5.65)	
Normal weight	0.80 (0.53, 1.21)	Reference	1.75 (1.38, 2.22)	
Overweight	0.28 (0.13, 0.63)	Reference	1.45 (1.02, 2.07)	
Obese	0.91 (0.40, 2.09)	Reference	1.58 (0.93, 2.70)	
Neonatal ward admission				0.293
Underweight	0.60 (0.29, 1.27)	Reference	0.71 (0.27, 1.89)	
Normal weight	0.80 (0.53, 1.21)	Reference	1.75 (1.38, 2.22)	
Overweight	0.28 (0.13, 0.63)	Reference	1.45 (1.02, 2.07)	
Obese	0.91 (0.40, 2.09)	Reference	1.58 (0.93, 2.70)	

Abbreviations: CI: confidence interval; OR: odds ratio; SGA, small-for-gestational age; LGA, large-for-gestational age. Values are odds ratios (95% confidence intervals). Adjusted for hospitals, maternal age, education, employment, average household income, ethnicity, parity, smoking before pregnancy, passive smoking during pregnancy, drinking during pregnancy, and gestational age at delivery.

Table S3. Association between gestational weight gain and pregnancy outcomes by maternal age

Outcome	Insufficient	Adequate	Excess	*P* for interaction
Cesarean delivery				0.031
<35 years	0.94 (0.80, 1.09)	Reference	1.28 (1.15, 1.43)	
≥35 years	1.07 (0.82, 1.38)	Reference	1.78 (1.44, 2.20)	
Low birthweight				0.784
<35 years	0.68 (0.38, 1.19)	Reference	0.72 (0.47, 1.11)	
≥35 years	0.98 (0.31, 3.14)	Reference	1.03 (0.40, 2.64)	
SGA				0.358
<35 years	1.39 (1.06, 1.82)	Reference	1.08 (0.87, 1.35)	
≥35 years	1.94 (1.15, 3.26)	Reference	1.31 (0.81, 2.12)	
Macrosomia				0.625
<35 years	0.75 (0.49, 1.15)	Reference	1.72 (1.36, 2.19)	
≥35 years	0.86 (0.46, 1.62)	Reference	1.84 (1.23, 2.76)	
LGA				0.943
<35 years	0.64 (0.43, 0.95)	Reference	1.66 (1.34, 2.05)	
≥35 years	0.67 (0.38, 1.18)	Reference	1.67 (1.17, 2.38)	
Neonatal ward admission				0.090
<35 years	0.93 (0.66, 1.32)	Reference	1.19 (0.93, 1.53)	
≥35 years	1.59 (0.91, 2.78)	Reference	1.98 (1.28, 3.07)	

Abbreviations: CI: confidence interval; OR: odds ratio; SGA, small-for-gestational age; LGA, large-for-gestational age. Values are odds ratios (95% confidence intervals). Adjusted for hospitals, prepregnancy BMI categories, education, employment, average household income, ethnicity, parity, smoking before pregnancy, passive smoking during pregnancy, drinking during pregnancy, and gestational age at delivery.

Table S4. The interaction between gestational weight gain and maternal age on the risk of cesarean delivery

		Cesarean delivery, n (%)	Model 1	Model 2
<35 years	Insufficient	310 (29.0)	1.04 (0.90, 1.21)	0.91 (0.78, 1.07)
	Adequate	1269 (28.1)	Reference	Reference
	Excess	1024 (37.0)	1.50 (1.36, 1.66)	1.37 (1.24, 1.53)
≥35 years	Insufficient	143 (41.7)	1.83 (1.46, 2.29)	1.60 (1.26, 2.03)
	Adequate	495 (39.2)	1.65 (1.45, 1.88)	1.48 (1.29, 1.70)
	Excess	385 (56.3)	3.29 (2.79, 3.87)	2.72 (2.27, 3.24)
P for interaction			0.031	0.034

Abbreviations: CI: confidence interval; OR: odds ratio; SGA, small-for-gestational age; LGA, large-for-gestational age. Values are odds ratios (95% confidence intervals). Model 1 was unadjusted model. Model 2 was adjusted for hospitals, prepregnancy BMI categories, education, employment, average household income, ethnicity, parity, smoking before pregnancy, passive smoking during pregnancy, drinking during pregnancy, and gestational age at delivery.

Table S5. Adjusted absolute risk differences for cesarean delivery by maternal age across gestational weight gain categories

		Absolute risk difference (%)	*P* value
Insufficient	<35 years	Reference	-
	≥35 years	12.5 (6.04, 18.97)	<0.001
Adequate	<35 years	Reference	-
	≥35 years	16.75 (12.39, 21.12)	<0.001
Excess	<35 years	Reference	-
	≥35 years	9.79 (6.34, 13.24)	<0.001

Table S6. Association between gestational weight gain and pregnancy outcomes controlling for adjust (n=10,638)

Outcome	Insufficient	Adequate	Excess
Cesarean delivery	0.94 (0.82, 1.08)	Reference	1.40 (1.27,1.54)
Low birthweight	0.72 (0.43, 1.19)	Reference	0.75 (0.51, 1.12)
SGA	1.47 (1.15, 1.87)	Reference	1.12 (0.91, 1.36)
Macrosomia	0.75 (0.52, 1.06)	Reference	1.88 (1.53, 2.32)
LGA	0.62 (0.45, 0.86)	Reference	1.75 (1.46, 2.11)
Neonatal ward admission	1.01 (0.75, 1.35)	Reference	1.36 (1.10, 1.69)

Abbreviations: CI: confidence interval; OR: odds ratio; SGA, small-for-gestational age; LGA, large-for-gestational age. Values are odds ratios (95% confidence intervals). Adjusted for hospitals, maternal age, prepregnancy BMI categories, education, employment, average household income, ethnicity, parity, smoking before pregnancy, passive smoking during pregnancy, drinking during pregnancy, gestational age at delivery, gestational diabetes mellitus, and hypertension in pregnancy.

Table S7. Association between gestational weight gain and pregnancy outcomes according to the 2009 Institute of Medicine recommendations (n=10,638)

Outcome	Insufficient	Adequate	Excess
Cesarean delivery	0.90 (0.81, 0.99)	Reference	1.37 (1.22, 1.54)
Low birthweight	0.76 (0.52, 1.12)	Reference	0.74 (0.46, 1.18)
SGA	1.59 (1.31, 1.94)	Reference	1.30 (1.01, 1.65)
Macrosomia	0.54 (0.42, 0.70)	Reference	1.40 (1.20, 1.75)
LGA	0.62 (0.50, 0.77)	Reference	1.54 (1.26, 1.88)
Neonatal ward admission	1.02 (0.82, 1.28)	Reference	1.37 (1.06, 1.76)

Abbreviations: CI: confidence interval; OR: odds ratio; SGA, small-for-gestational age; LGA, large-for-gestational age. According to the 2009 Institute of Medicine recommendations, total weight gain during pregnancy was classified into insufficient, adequate and excessive if it was below, within, or above the recommendations as follows: 12.5-18 kg (prepregnancy BMI< 18.5 kg/m^2), 11.5-16 kg (prepregnancy BMI: 18.5–24.9 kg/m^2), 7-11.5 kg (prepregnancy BMI: 25.0–29.9 kg/m^2), and 5-9 kg (prepregnancy BMI $\geq$ 30.0 kg/m^2).

Values are odds ratios (95% confidence intervals). Adjusted for hospitals, maternal age, prepregnancy BMI categories, education, employment, average household income, ethnicity, parity, smoking before pregnancy, passive smoking during pregnancy, drinking during pregnancy, and gestational age at delivery.

Table S8. Association between rate of gestational weight gain and pregnancy outcomes according to the 2009 Institute of Medicine recommendations (n=10,638)

Outcome	Insufficient	Adequate	Excess
Cesarean delivery	0.94 (0.82, 1.07)	Reference	1.37 (1.24, 1.51)
Low birthweight	1.23 (0.77, 1.97)	Reference	1.06 (0.72, 1.57)
SGA	1.05 (0.79, 1.38)	Reference	0.98 (0.80, 1.19)
Macrosomia	1.19 (0.85, 1.67)	Reference	2.13 (1.68, 2.71)
LGA	0.72 (0.50, 1.04)	Reference	1.94 (1.59, 2.37)
Neonatal ward admission	0.65 (0.47, 0.89)	Reference	1.30 (1.04, 1.62)

Rate of gestational weight gain in the second and third trimesters was classified as insufficient, adequate and excess if it was below, within, or above the recommendation for weight gain during pregnancy period proposed by the National Health Commission of the People's Republic of China (0.37-0.56 kg/weeks [prepregnancy BMI< 18.5 kg/m^2], 0.26-0.48 kg/weeks [prepregnancy BMI: 18.5–23.9 kg/m^2], 0.22-0.37 kg/weeks [prepregnancy BMI: 24.0–27.9 kg/m^2], and 0.15-0.30 kg/weeks [prepregnancy BMI ≥ 28.0 kg/m^2].

Values are odds ratios (95% confidence intervals). Adjusted for hospitals, maternal age, prepregnancy BMI categories, education, employment, average household income, ethnicity, parity, smoking before pregnancy, passive smoking during pregnancy, drinking during pregnancy, and gestational age at delivery.
